# Supplementary material for: Prolonged Extracorporeal Circulation Leads to Inflammation and Higher Expression of Mediators of Vascular Permeability Through Activation of STAT3 Signaling Pathway in Macrophages
Source: Int J Mol Sci. 2024 Nov 19;25(22):12398. doi: 10.3390/ijms252212398 (PMC11594647; doi:10.3390/ijms252212398)
Supplement: Supplementary file 1 [file ijms-25-12398-s001.zip › ijms-3207267-supplementary.pdf]

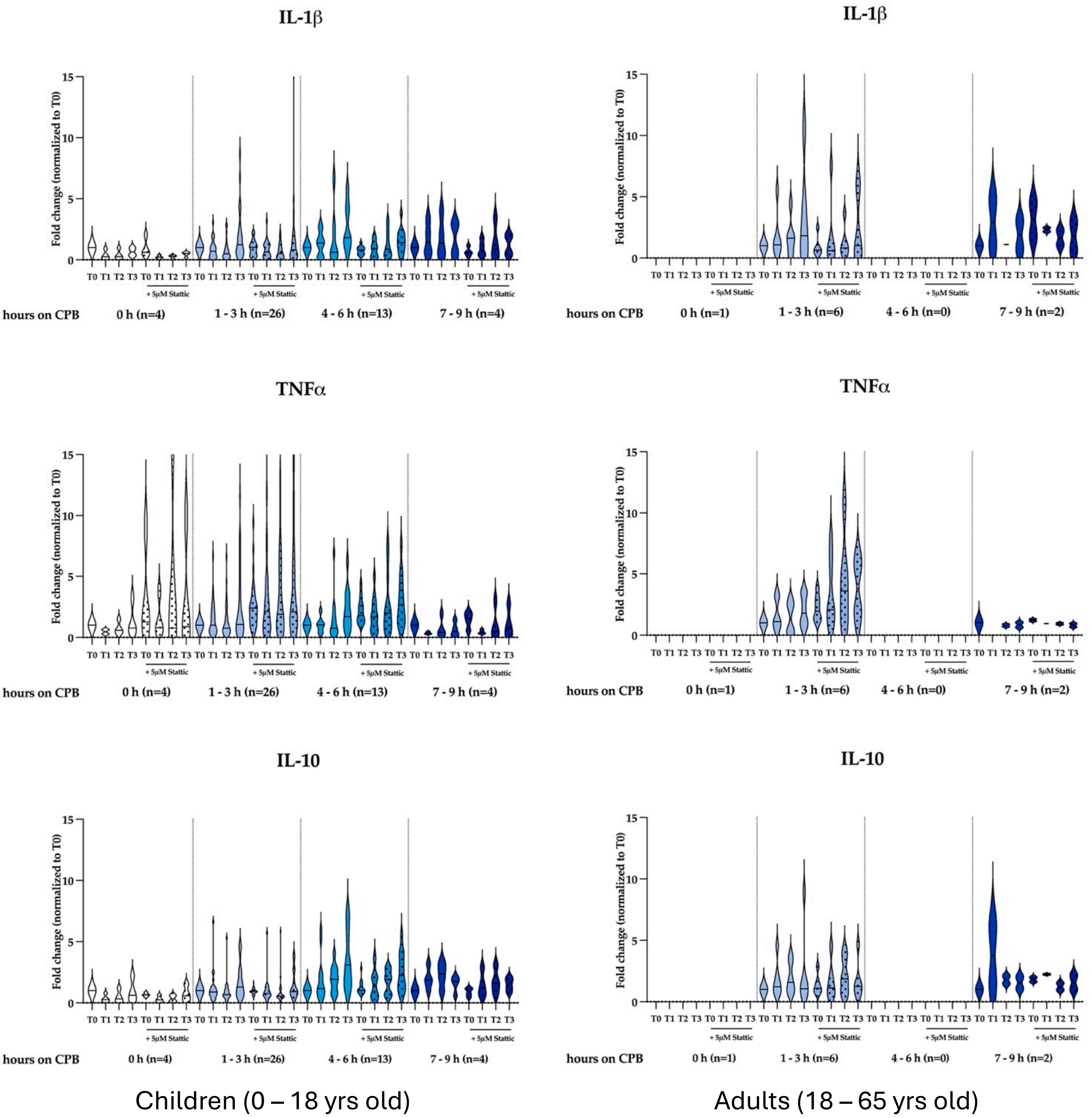

Figure S1. Effects of Stattic on cytokines expressions IL-1β, TNFα and IL-10 in THP-1 macrophages *in vitro* stimulated with serum samples from patients undergoing cardiac surgery without and with CPB. The patient cohort was classified into four distinct groups based on duration of CPB as followed: 0h (n=5), 1-3 h (n=32), 4-6 h (n=13) and 7-9 h (n=6). Data from 56 patients were also sub-grouped into Children (0 – 18 yrs old, n=47) and Adults (18 – 65 yrs old, n=9) and are represented as violin plots.

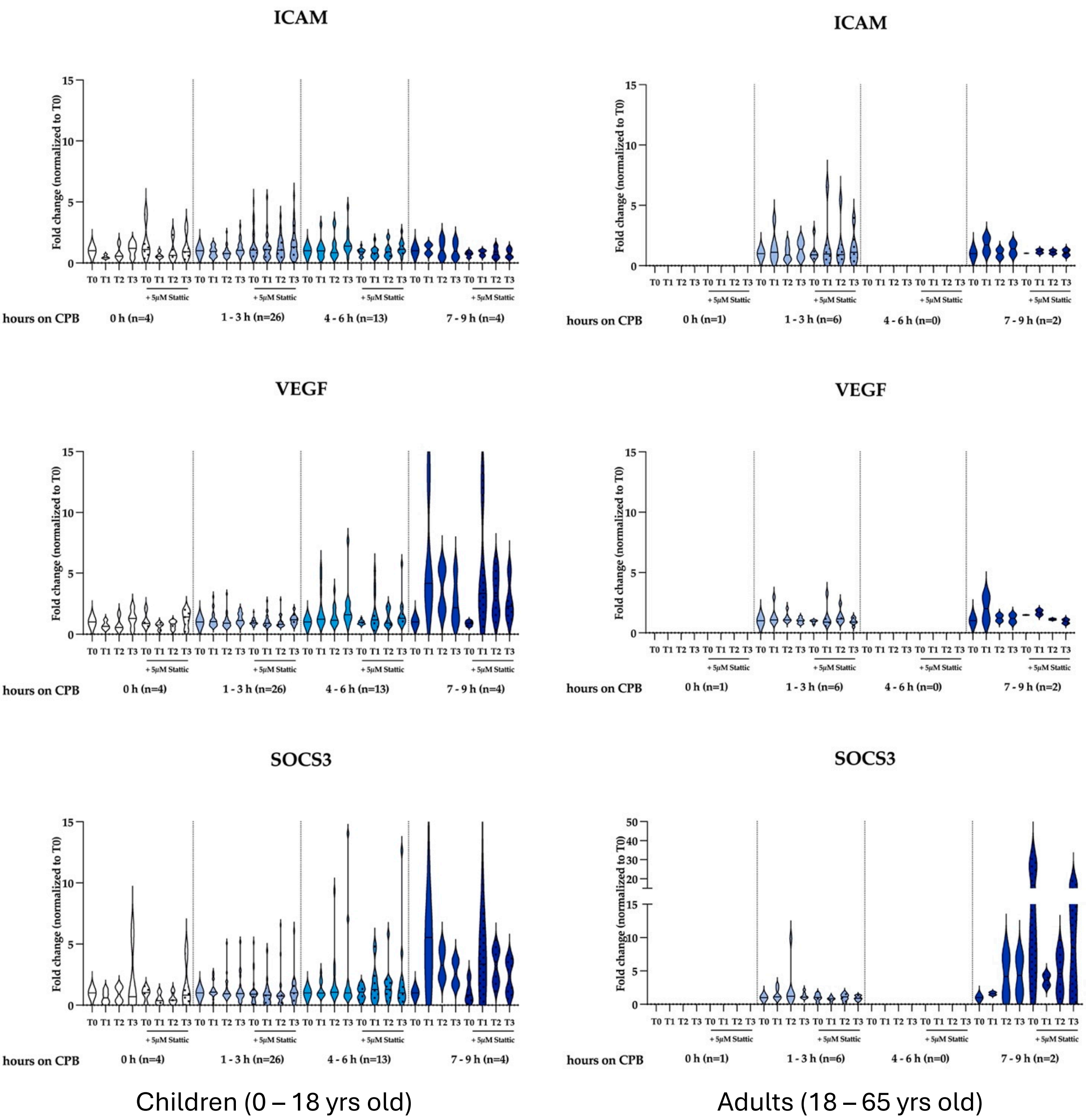

Figure S2. Effects of Stattic on mediators of vascular permeability ICAM, VEGF and the inhibitor of STAT3 activation (SOCS3) expressions in THP-1 macrophages *in vitro* stimulated with serum samples from patients undergoing cardiac surgery without and with CPB. The patient cohort was classified into four distinct groups based on duration of CPB as followed: 0h (n=5), 1-3 h (n=32), 4-6 h (n=13) and 7-9 h (n=6). Data from 56 patients were also sub-grouped into Children (0 – 18 yrs old, n=47) and Adults (18 – 65 yrs old, n=9) and are represented as violin plots.
